# A mutagenesis analysis of Tim50, the major receptor of the TIM23 complex, identifies regions that affect its interaction with Tim23

**DOI:** 10.1038/s41598-018-38353-1

**Published:** 2019-02-14

**Authors:** Dana Dayan, May Bandel, Umut Günsel, Inbal Nussbaum, Gali Prag, Dejana Mokranjac, Walter Neupert, Abdussalam Azem

**Affiliations:** 1 Department of Biochemistry and Molecular Biology, The George S. Wise Faculty of Life Sciences, Tel Aviv University, Tel Aviv, 69978 Israel; 2 Biomedical Center Munich - Physiological Chemistry, LMU Munich, 82152 Martinsried, Germany; 3 Max Planck Institute of Biochemistry, 82152 Martinsried, Germany

## Abstract

Maintenance of the mitochondrial proteome depends on import of newly made proteins from the cytosol. More than half of mitochondrial proteins are made as precursor proteins with N-terminal extensions called presequences and use the TIM23 complex for translocation into the matrix, the inner mitochondrial membrane and the intermembrane space (IMS). Tim50 is the central receptor of the complex that recognizes precursor proteins in the IMS. Additionally, Tim50 interacts with the IMS domain of the channel forming subunit, Tim23, an interaction that is essential for protein import across the mitochondrial inner membrane. In order to gain deeper insight into the molecular function of Tim50, we used random mutagenesis to determine residues that are important for its function. The temperature-sensitive mutants isolated were defective in import of TIM23-dependent precursor proteins. The residues mutated map to two distinct patches on the surface of Tim50. Notably, mutations in both patches impaired the interaction of Tim50 with Tim23. We propose that two regions of Tim50 play a role in its interaction with Tim23 and thereby affect the import function of the complex.

## Introduction

Mitochondria import the majority of their proteins from the cytosol, a process mediated by several sophisticated protein translocation machineries. Almost all mitochondrial precursor proteins cross the outer membrane through the TOM complex (Translocase of the Outer Mitochondrial Membrane). Subsequent cooperation of the TOM complex with additional import machineries facilitates the sorting and assembly of precursor proteins in the various mitochondrial compartments^1–3^.

Mitochondrial precursor proteins that contain positively-charged amino terminal presequences are recognized and handled by the TIM23 complex (Translocase of the Inner Mitochondrial Membrane). The TIM23 complex imports essentially all proteins targeted to the matrix and some proteins targeted to the inner membrane and the inter membrane space (IMS)^1,2^. Interestingly, two outer membrane proteins, Om45 and Mcp3, are inserted into the outer membrane by a novel mechanism that also involves the TIM23 complex^3–5^.

The core of the TIM23 complex is composed of three essential inner membrane proteins Tim23, Tim50 and Tim17. Tim23 fulfills two important functions: it binds presequences in the IMS and, likely together with Tim17, forms the membrane potential- dependent translocation channel^6–8^. Tim23 and Tim17 are tightly associated with each other, forming a platform to which other components of the translocase are recruited^9,10^. Two additional, membrane-embedded subunits of the TIM23 complex are the nonessential proteins Mgr2 and Tim21 that have a role in the lateral insertion into the mitochondrial inner membrane and also appear to link the TIM23 complex with the respiratory chain complexes^11–13^. In addition to these five subunits which enable the initial translocation step, the TIM23 complex contains a matrix-exposed import motor (also known as PAM - Presequence translocase Associated Motor). The import motor, consisting of the Hsp70 chaperone and its regulating subunits, completes translocation across the inner membrane in an ATP-dependent manner^14^.

Tim50 is anchored in the inner membrane with a single transmembrane segment and exposes a large domain in the IMS. It functions as the primary presequence receptor for incoming precursors that directs them to the channel in the inner membrane^15–20^. Tim50 also appears to play a role in maintaining the permeability barrier of the mitochondrial inner membrane^21^. The IMS domain of yeast Tim50 can be divided into the core domain (amino acid residues 164–361) and the presequence-binding domain (PBD - amino acid residues 395–476). The core domain was crystallized and demonstrated to interact with Tim23 and Tim21^22^. The PBD can be crosslinked to presequence peptides and may also mediate the interaction with Tom22, a receptor subunit of the TOM complex^15–18,22,23^. PBD is not the only presequence-binding domain on yeast Tim50. Also the core domain was shown to interact on its own with presequences^17^. Though each domain exhibited a relatively high affinity for presequences, a single interacting site is obviously not sufficient to support the function of Tim50^17^.

Transfer of precursor proteins from the TOM complex to the translocation channel of the TIM23 complex in the inner membrane strictly depends on the interaction between IMS-exposed domains of Tim50 and Tim23^16,19,20,24–28^. Mutations of Tim23 and Tim50 that disrupt the interaction between the two proteins lead to temperature-sensitive (*ts*) growth defects and impair translocation of proteins through the TIM23 complex^20,24^. Thus, the interaction of Tim50 with Tim23 is important both for its receptor function and for the efficient transfer of precursor proteins from the TOM complex to the TIM23 complex^20,24^.

Mutational analysis of the IMS domain of Tim50 that mediates its interaction with Tim23 has been the subject of two studies^16,24^. These studies reached different conclusions regarding the amino acid residues that are important for the interaction^16,24^. The first study reported that the triple mutant, L279S/L282S/L286S, exhibited a *ts* growth phenotype and displayed impaired interaction with Tim23^24^. In the second study, the crystal structure of the core domain of Tim50 was solved. It showed that these three residues are buried deep in the hydrophobic core of Tim50, potentially causing destabilization of the molecule^16,24^. In the same report, the authors suggested that two amino acid residues of Tim50, R214 and K217, mediate the Tim23-Tim50 interaction, since mutating these residues resulted in reduced binding of the two proteins^16^.

In the present study, we used random mutagenesis, as an unbiased approach, to gain deeper insight into the interactions of Tim50 with its partner proteins. Notably, all of the identified Tim50 mutants exhibited impaired interaction between Tim50 and Tim23. Our results indicate that two highly conserved but distinct regions of the IMS-exposed domain of Tim50 play a role, directly or indirectly, in the interaction of the protein with Tim23 and thereby are crucial for the function of the TIM23 complex.

## Results

### Isolation and characterization of temperature-sensitive Tim50 mutants

**Figure 1:**
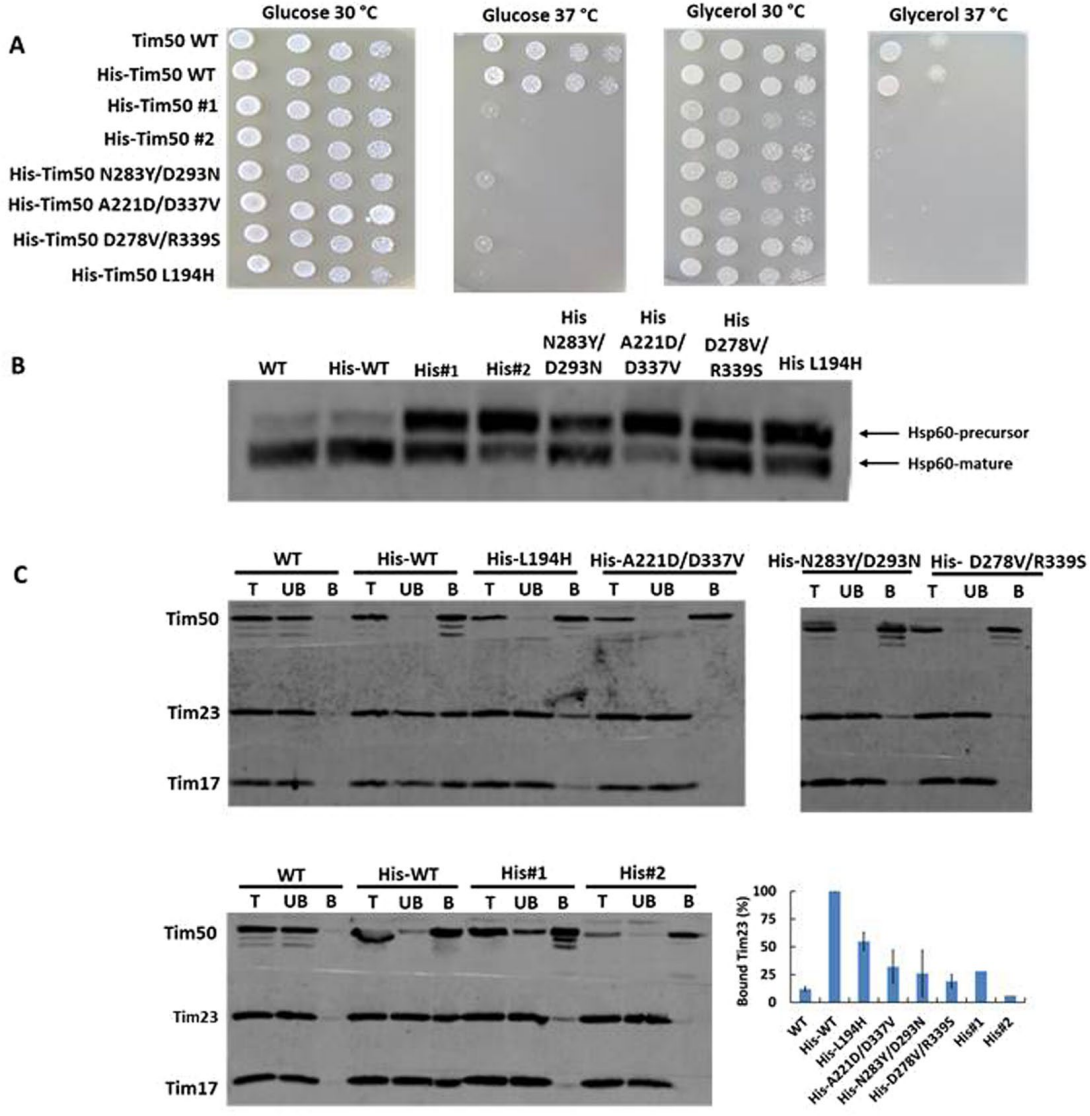
Isolation and characterization of temperature sensitive mutants of Tim50. (**A**) Serial dilutions of Tim50 WT and mutant cells were grown for 5 days at the indicated temperature on SCD (-Leu) and SCG plates. (**B**) Accumulation of the precursor form of Hsp60 in wild type and mutant cells was analyzed by SDS-PAGE and immunoblot with Hsp60 antibodies, as described under Methods. (**C**) Mitochondria isolated from WT cells, or from cells expressing indicated His tagged versions of Tim50 were solubilized with 1% digitonin and incubated with Ni-NTA agarose beads at 4 °C. Following washing steps, specifically bound proteins were eluted with Laemmli buffer containing 300 mM imidazole. Samples were analyzed by SDS-PAGE and immunoblotting with Tim50, Tim17 and Tim23 antibodies. T, UB, 10% of total and unbound material. B, 100% of bound material. Binding of Tim23 was quantified from three independent experiments for His-tagged WT, L194H, A221D/D337V, N283Y/D293N and D2787V/R339S mitochondria and from two for His#1 and His#2 mitochondria. The efficiency of Tim23 binding to Ni-NTA agarose beads with mitochondria isolated from cells expressing His-tagged but otherwise WT version of Tim50 was set to 100%.

To identify residues of Tim50 that affect its function in an unbiased way, we sought to generate mutations that cause temperature-sensitive (*ts*) growth of yeast cells. To this end, we generated a library of random mutants on the sequence of the mature form of Tim50. Six out of approximately 2000 screened colonies showed a *ts* growth phenotype. The amino acid residues altered in the individual mutants are listed in Table 1. Notably, all of the mutants harbored substitutions in more than one amino acid residue. In an attempt to identify residues that are directly responsible for the *ts* phenotype, we first analyzed mutants #1 and #2 that contained five substitutions each. We chose to study the most conserved and reasonably surface-exposed residues (V274 and R218 in these mutants, respectively). In addition, we studied residue L202 that was mutated in both mutants. The growth phenotypes of all single mutants chosen were indistinguishable from that of wild type (WT) cells (data not shown) and therefore, mutants #1 and #2 were kept in their original form. With mutants #3 to #6, we cloned all possible combinations of amino acid residues derived from the original mutants. For example, with mutant #4, only the combination of mutations affecting both residues N283 and D293 led to temperature-sensitive growth (Supplementary Fig. S1). Based on such observations, we focused our further experiments on the two mutants that contained five substitutions (mutants #1 and #2), on three double mutants, D278V/R339S (derived from mutant #3), N283Y/D293N (derived from mutant #4) and A221D/D337V (derived from mutant #5), and on one single mutant L194H (derived from mutant #6) (Table 1). All mutants exhibited strong growth defects both on glucose- and on glycerol-containing media at 37 °C (Fig. 1A).

**Table 1:** Temperature-sensitive mutants identified in the screen and the derived mutants.

Original mutants		Derived mutants	
Name	Mutations	Name	Mutations
# 1	L202V,F237L,Y261S,V274E,Y299S		
# 2	L202M,R218G,S298R,L362S,S386F		
# 3	P175H,D278V,R339S	D278V/R339S	D278V,R339S
# 4	N283Y,D293N,E439G	N283Y/D293N	N283Y,D293N
# 5	A221D,M242K, D337V	A221D/D337V	A221D,D337V
# 6	L194H,E415D	L194H	L194H

We examined whether the various *ts* mutants accumulate the precursor form of the mitochondrial matrix protein Hsp60, an indicator of the mitochondrial protein import defect^9^. To this end, yeast cells were grown at 37 °C and total cell extracts were analyzed by SDS-PAGE followed by immunoblotting with antibodies against Hsp60. Increased levels of the precursor form of Hsp60 were observed in all *ts* mutants examined (Fig. 1B). The import defects observed *in vivo* were also seen when *in vitro* import into isolated mitochondria of various TIM23 substrates was analyzed (Supplementary Fig. S2). Taken together, these results suggest that impaired protein import into mitochondria is the cause of the observed growth defect.

We reasoned that the *ts* mutations may impair the interaction of Tim50 with Tim23. Mitochondria, expressing either WT or His-tagged versions of Tim50, were solubilized with digitonin and subjected to a pull down assay using Ni-NTA agarose beads. When mitochondria harboring His-tagged but otherwise wild type Tim50 were analyzed, as expected, in addition to the tagged protein two other core subunits, Tim23 and Tim17, were found in the bound fraction. Less efficient binding of Tim23 and Tim17 to Ni-NTA agarose beads was observed in mitochondria from the *ts* mutants (Fig. 1C). The reduced binding was not due to the decreased levels of the proteins as an analysis of the mitochondrial profiles revealed no major differences between WT and mutant mitochondria (Supplementary Fig. S3). None of the proteins bound to Ni-NTA agarose beads when WT mitochondria were analyzed, demonstrating the specificity of the assay.

We conclude that the observed growth and import defects are due to the impaired interaction between Tim50 and Tim23.

### Mapping of the identified mutations on the structure of Tim50

**Figure 2:**
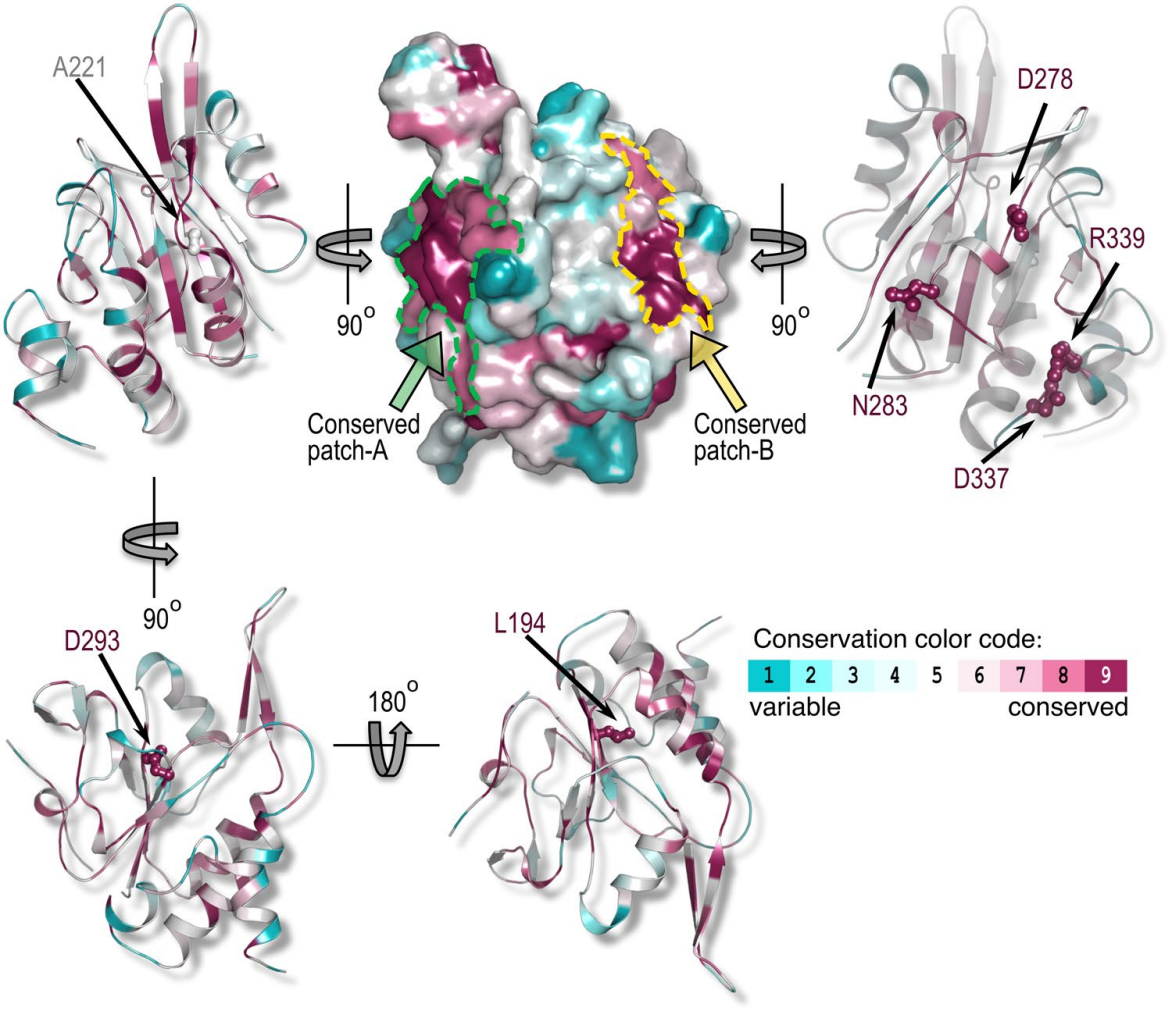
Mapping of the residues identified in the *ts* mutants on the structure of Tim50. Residues mutated in the Tim50 mutants identified in this study were projected on a three dimensional structure of Tim50 (PDB entry 4qqf). Residues are color coded based on their conservation level as calculated by Consurf^31^ (see color code ruler; cyan, most variable; maroon, most conserved). Mutated residues are rendered as ball-and-stick models. Two highly conserved patches (designated as patch-A and patch-B) are shown in the top middle panel. The molecule is rotated by +/− 90 degrees on the Y-axis in order to visualize the positions of the individual residues in these patches. The conserved residues D293 and L194, which are buried in the core of the protein and are not part of the identified patches, are shown in the bottom panels.

The availability of the crystal structure of the core domain of Tim50^22^ enabled us to ask whether the mutations identified provide a means to define the binding site on Tim50 for Tim23. Except for residues L362 and S386 that are not present in the crystallized domain of Tim50, all other residues altered in the mutants identified could be mapped on the available structure. In mutants #1 and #2, mutations affected conserved residues that are buried within the hydrophobic core of the protein as well as non-conserved, surface-exposed residues (data not shown). Thus, the observed phenotypes of these mutants are likely the consequence of the mutations in the hydrophobic core of Tim50 that led to a profound alteration in the protein structure. Similarly, residue L194 mutated in the only single mutant identified, is present in the hydrophobic core of Tim50 (Fig. 2) and its mutation likely also led to a significant destabilization of the structure.

All residues mutated in the double mutants N283Y/D293N, A221D/D337V and D278V/R339S are highly conserved during evolution, with the exception of A221. They are also reasonably well surface exposed, except for A221 and D293. With the exception for D293, they all map to two conserved patches on the surface of Tim50, which we named patch A and patch B. Interestingly, within each mutant, the two altered residues are positioned at a considerable distance from each other, making it unlikely that they constitute a continuous, single Tim23-binding site (Fig. 2). We therefore asked whether double mutations are required to interfere with Tim23 binding or single mutations would already suffice.

### Single mutations are sufficient to impair the Tim23-Tim50 interaction

**Figure 3:**
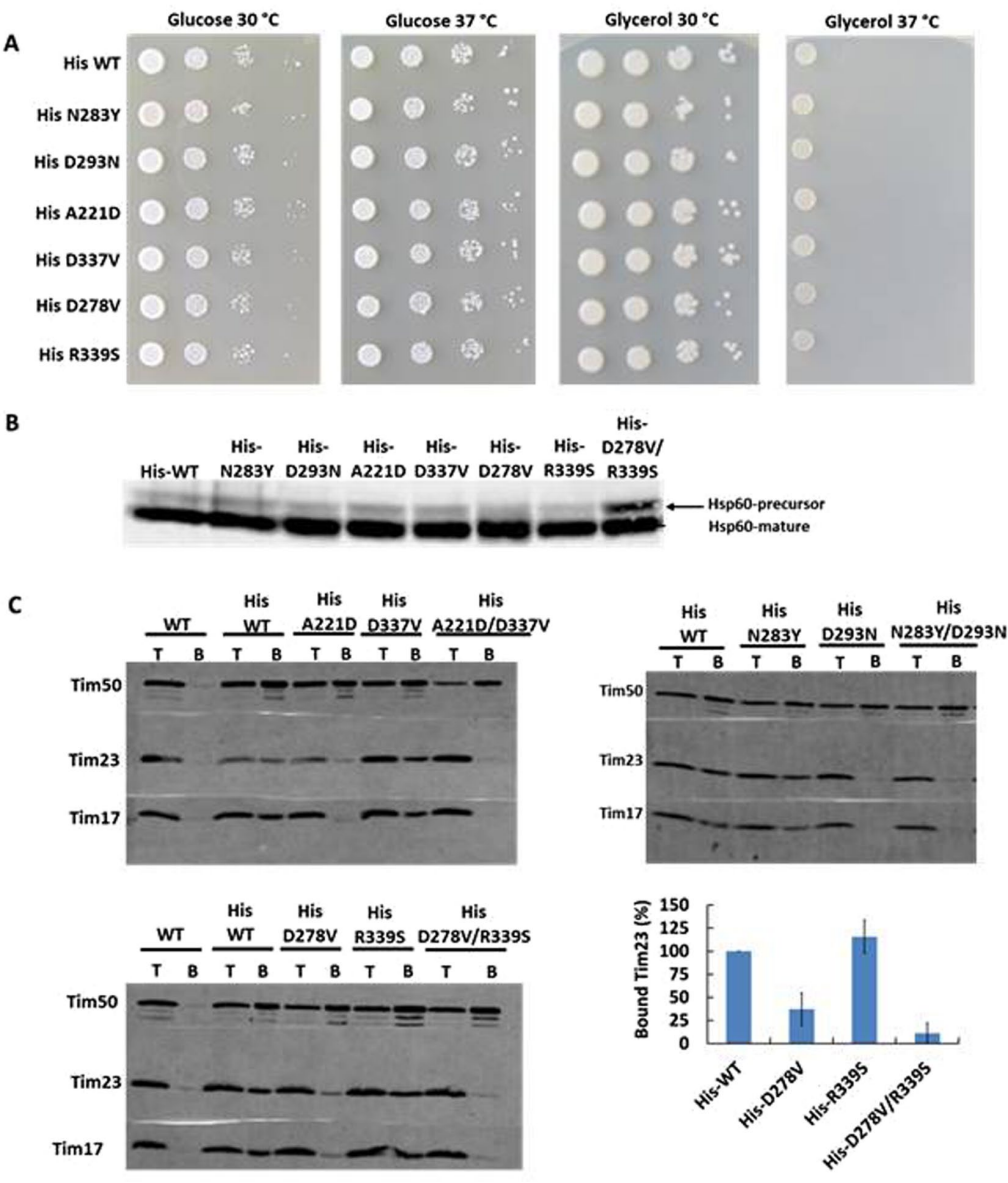
Analysis of Tim50 single mutants. (**A**) Serial dilutions of Tim50 WT and mutant cells were grown for 5 days at the indicated temperature on SCD (-Leu) and SCG plates. (**B**) Accumulation of the precursor form of Hsp60 in wild type and mutant cells was analyzed by SDS-PAGE and immunoblot with Hsp60 antibodies, as described under Methods. (**C**) Tim23-Tim50 interaction in the indicated strains was analyzed using pull down assay with Ni-NTA agarose beads essentially as described in Fig. 1C. T, 10% of total material. **B**, 100% of bound material. As an example, binding of Tim23 to Ni-NTA agarose beads using D278V/R339S double mutant and its corresponding single mutants was quantified as described in Fig. 1C.

We analyzed the six positions mutated in N283Y/D293N, A221D/D337V and D278V/R339S double mutants. The six single mutants derived from these double mutants displayed normal growth (Fig. 3A). They also showed no obvious import defect *in vivo* (Fig. 3B) and *in vitro* for most of the TIM23 substrates analyzed (Supplementary Fig. S2). Notably, when Tim50-Tim23 interaction was examined in a Ni-NTA pull-down assay, one single mutant derived from each double mutant showed impaired interaction with Tim23. The single mutants with impaired Tim50-Tim23 interaction were A221D, D278V and D293N. The remaining three mutants exhibited interactions that were similar to that of the WT protein (Fig. 3C).

**Figure 4:**
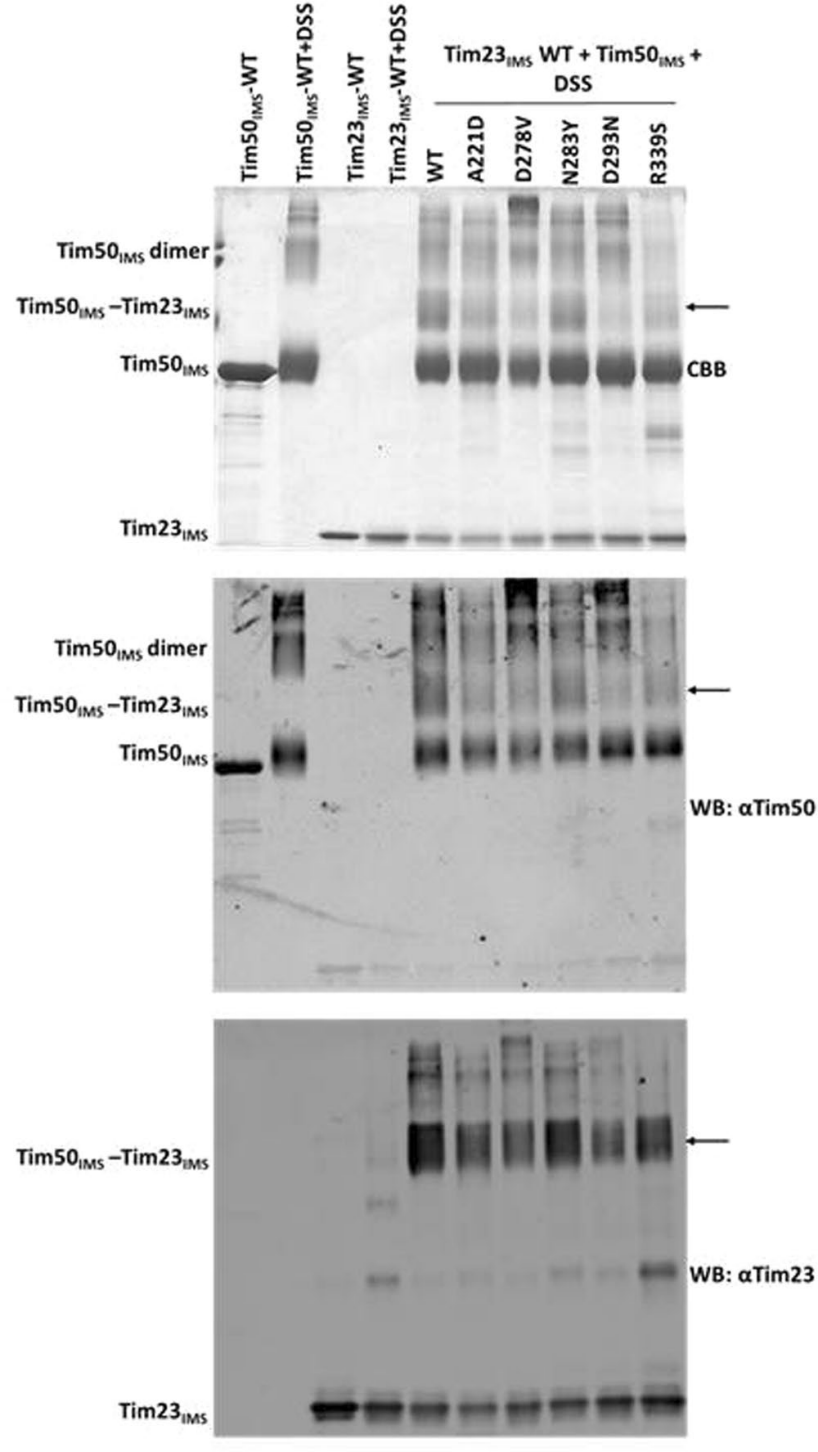
Analysis of the Tim23-Tim50 interaction *in vitro*. Recombinantly expressed and purified versions of Tim50_IMS_ were incubated with Tim23_IMS_ in the presence of 1 mM DSS, as indicated. Cross-linking adducts were separated by SDS-PAGE and analyzed by staining with Coomassie Brilliant Blue (CBB) (top panel) or subjected to immunoblotting with antibodies raised against Tim50 (middle panel) and Tim23 (bottom panel). Arrows indicate the Tim23-Tim50 crosslink. Mutant D337V was not analyzed as it was largely insoluble after recombinant expression. The R339S mutant was partially degraded, suggesting that the recombinantly expressed mutant protein is less stable.

We further used an *in vitro* crosslinking assay that we previously established to analyze the interaction between Tim23 and Tim50^20^. Briefly, the recombinantly expressed and purified IMS domains of Tim50 and Tim23 were incubated together in the presence of the crosslinker DSS followed by the analysis of the crosslinking products using SDS-PAGE (Fig. 4). We observed no difference in the crosslinking efficiency between Tim50_IMS_ and Tim23_IMS_ when the Tim50 N283Y and Tim50 R339S mutants were analyzed. In contrast, mutations A221D, D278V and D293N reduced the efficiency of the crosslinking between Tim50_IMS_ and Tim23_IMS_ (Fig. 4). The identity of the Tim50-Tim23 crosslink, shown by CBB staining in Fig. 4, top panel, was confirmed by immunoblotting using antibodies against Tim50 (Fig. 4, middle panel) and Tim23 (Fig. 4, bottom panel).

Taken together, both *in organello* and *in vitro* analyses suggest that A221D, D278 and D293 are involved, directly or indirectly, in the Tim23-Tim50 interaction. Thus, yeast cells can tolerate, to a limited degree, destabilization of the Tim23-Tim50 interaction in a manner that does not affect their ability to grow at higher temperatures and does not severely affect import efficiency of mitochondrial proteins.

### Two patches on Tim50 are involved in its binding to Tim23

**Figure 5:**
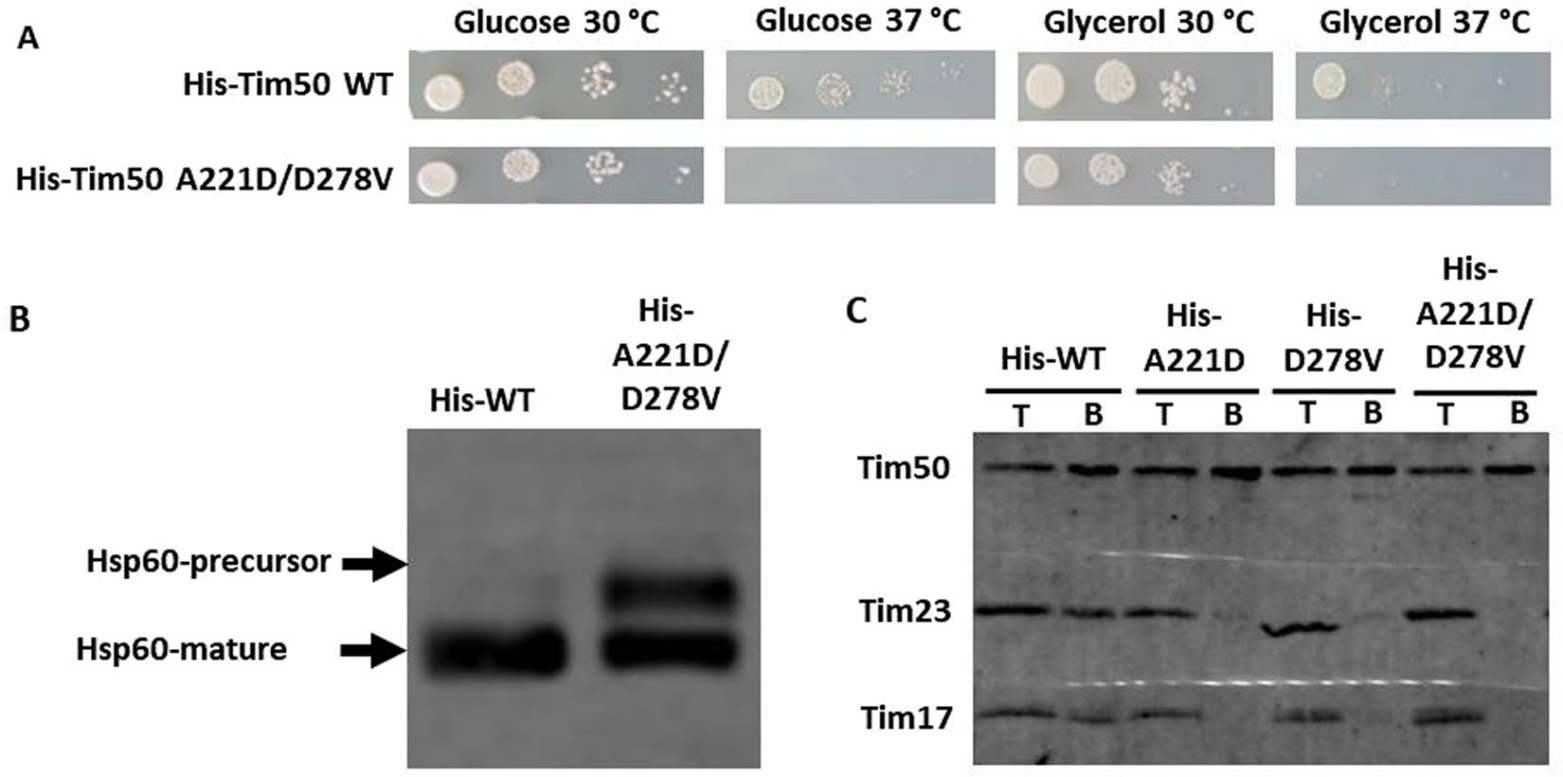
Characterization of the A221D/D278V mutant. (**A**) Cell growth, (**B**) Hsp60 precursor accumulation *in vivo* and (**C**) analysis of the Tim23-Tim50 interaction were carried out essentially as described in Fig. 1.

Is it possible that the two distant patches on Tim50 play a role in its interaction with Tim23? If so, then mutating two residues together that each affect Tim23 binding, one from each patch, should destabilize the Tim50-Tim23 interaction even further so that an obvious growth and import phenotypes should become visible. To test this, we created a strain that carries the double mutation A221D, D278V (Fig. 5). The mutant indeed did not grow at 37 °C (Fig. 5A), the precursor form of Hsp60 accumulated *in vivo* (Fig. 5B) and the interaction with Tim23 was abolished (Fig. 5C).

In summary, these results suggest that the two surface-exposed patches on Tim50 are involved, directly or indirectly, in its interaction with Tim23.

## Discussion

In the present study, we set up a system for identifying temperature-sensitive mutants of Tim50 as a tool for revealing functionally important residues in this protein. We show that in all mutants identified in the screen, the interaction of Tim50 with Tim23 was impaired and import of mitochondrial proteins via the TIM23 complex was reduced.

**Figure 6:**
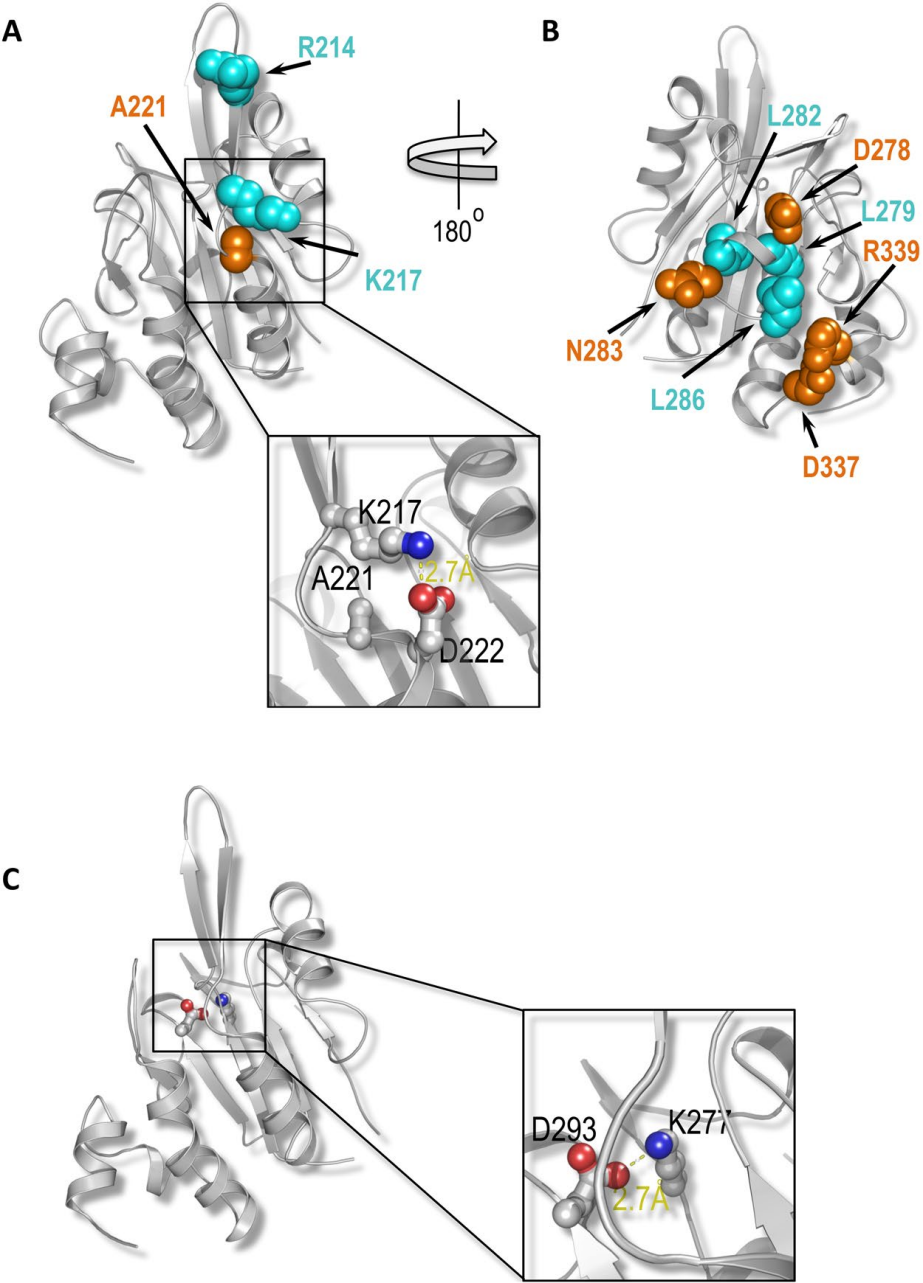
Molecular insight into regions of Tim50 involved in interaction with Tim23. (**A**) Residue A221 (orange) identified in the screen here is close to residues R214 and K217 (both in cyan) that were previously implicated in Tim50 interaction with Tim23 by Qian *et al*.^16^. The inset depicts the electrostatic interaction between K217 and D222 that is likely changed by A221D mutation, see text for details. Amino group in the lysine side chain is shown in blue and two oxygen atoms in the carboxyl group of the aspartate side chain are shown in red. (**B**) Residues D278, N283, D337 and R339 (all in orange) mutated in *ts* mutants identified in the present screen map closely to residues L279, L282 and L286 (all in cyan) previously implicated in Tim50-Tim23 interaction by Tamura *et al*.^24^. The model shown in (**B**) is rotated by 180° relative to the model shown in (**A**). The orientations shown in panels A and B correspond to the orientations shown in the top right and top left panels, respectively, in Fig. 2. (**C**) Mapping of residue D293 identified here in the screen on the structure of Tim50. The inset depicts the electrostatic interaction between K277 and D293 that is likely affected by D293N mutation, see text for details. Amino group in the lysine side chain is shown in blue and two oxygen atoms in the carboxyl group of the aspartate side chain are shown in red.

Based on the crystal structure, the residues comprising the three double mutants, N283Y/D293N, A221D/D337V and D278V/R339S, map to two patches. Surface conservation analysis showed that these two patches are highly conserved, further supporting the notion of their importance (Fig. 2). Intriguingly, the two patches map closely to the residues previously identified by Tamura *et al*. and Qian *et al*.^16,24^ (Fig. 6). Thus, the unbiased screen performed here reconciles the seemingly conflicting data reported previously. Apparently, both patches play an important role in Tim50-Tim23 interaction. In patch A, A221 is located within the region close to residues R214 and K217 identified by Qian *et al*.^16^ (Fig. 6A). How can the A221D mutation trigger dissociation of the Tim23-Tim50 complex? The available crystal structure suggests that residue D222 forms a salt bridge with the nearby residue K217 (Fig. 6A). A221 is not a very well conserved residue, however, its mutation to an aspartate introduces an additional negative charge in the region, potentially causing a repulsion between two negative charges and thereby changing the surface of this region into a conformation that is not conducive to Tim23 binding (Fig. 6A). On the opposite side of the Tim50 molecule, in patch B, we mapped residues D278, N283, D337 and R339 (Fig. 6B). They are in proximity of residues L279, L282 and L286 identified by Tamura *et al*.^24^. Among these residues, D278 may participate directly in the interaction of Tim50 with Tim23, whereas D337 and R339 appear to play a minor role in the interaction. Deep in a groove between the two patches, we identified residue D293 (Fig. 6C). The available crystal structure suggests that it forms a salt bridge with K277. Removing the negative charge by the D293N mutation possibly leads to an altered conformation of Tim50 that is incompatible with Tim23 binding.

Surprisingly, single mutations A221D, D278V and D293N, that destabilize the Tim23-Tim50 interaction, are well tolerated by yeast cells. Destabilization of Tim23-Tim50 interaction in these mutants is obvious upon solubilization of mitochondria and in crosslinking *in vitro* but not upon growth phenotype examination. Apparently, only drastic negative effects on the Tim23-Tim50 interaction will lead to growth and import defects *in vivo*, suggesting a high degree of plasticity of Tim23-Tim50 interaction. A possible explanation may be in the way we assessed the Tim50-Tim23 interaction, since we observed a severe destabilization of their interaction upon solubilization of mitochondria. Considering how unstable the TIM23 complex is after solubilization even with the mildest detergents available, this method possibly overestimates the impairment of Tim50-Tim23 interaction. In intact mitochondria, the native lipid environment and the other subunits of the complex may stabilize this interaction. Such additional, potentially stabilizing factors would be also absent in our crosslinking experiments performed with recombinant proteins.

How can two distinct patches that are far apart from each other play a role in the Tim50 interaction with Tim23? The ~10 kDa IMS domain of Tim23 is intrinsically disordered^20,29^ and, conceivably, due to its conformational flexibility, it can associate with multiple patches on Tim50. This view is supported by the fact that an NMR study identified several Tim50 binding sites on Tim23^25^. Furthermore, two different Tim23 subunits of the TIM23 complex could occupy two distinct binding sites on Tim50. The presence of Tim23 dimers potentially would speak for this scenario^20^. Two binding sites may also be used at different stages of protein translocation. The latter possibility is particularly appealing in the light of the many steps that need to take place in the IMS during the transfer of the precursor protein from the *trans* side of the TOM complex to the translocation channel of the TIM23 complex in the inner membrane.

Taken together, the data presented here reconcile two previous seemingly contradictory reports^16,24^ and indicate that two highly conserved but distinct patches of the IMS-exposed domain of Tim50 are involved, directly or indirectly, in the interaction of Tim50 with Tim23 and thereby are crucial for the function of the TIM23 complex.

## Methods

### Plasmids, yeast strains and cell growth

A pGEM4 plasmid containing the sequence encoding the mature Tim50 was used as template for random and site directed mutagenesis. The resulting mutated nucleotide sequence was cloned into the centromeric yeast plasmid pRS315 containing the presequence of Tim50 followed by the His-tag, under the control of the endogenous *TIM50* promoter and 3′UTR. The plasmids were transformed into a Tim50 plasmid shuffling strain. This strain carries a chromosomal deletion of Tim50 and a WT copy of *TIM50* on a plasmid carrying a URA marker^30^.

Cells were grown on synthetic media lacking leucine, containing 2% glucose (SCD (-Leu)) or on synthetic media containing 3% glycerol (SCG). Yeast strains for isolation of mitochondria were grown in lactate medium containing 0.1% glucose at 24 °C.

### Random mutagenesis and screening

A library of random Tim50 mutants was generated through error prone PCR on the sequence of mature Tim50, using MutazymeII DNA polymerase (Agilent technologies) according to the manufacturer’s instructions. The reaction was carried out with forward primer 5′ GGATCCCAAAAAGAAACAAAAGACGAC3′, and reverse primer 5′AAGCTTTTATTT GGATTCAGCAATCTTC3′ on 300 ng target DNA (total reaction volume 50 μl). The resulting PCR products were digested with BamHI and HindIII and were ligated into the pRS315 vector described above. The Tim50 plasmid shuffling strain described above was transformed with the library. Transformants were patched on an SCD plate lacking uracil and leucine and were then replica plated on medium containing 5-fluoroorotic acid (5-FOA) to select for colonies that express only Tim50 encoded by pRS315 and have lost the URA plasmid. In order to find temperature sensitive mutants, the 5-FOA plates were replica plated on two SCD (-Leu) plates, one for the examination of growth at 30 °C and the other at 37 °C. Colonies exhibiting temperature sensitive growth were isolated and the coding region of Tim50 was sequenced.

### Site-directed mutagenesis

Site-directed mutants were created according to the protocol of Agilent technologies, using the primers listed in Supplementary Table S1.

### Hsp60 precursor accumulation

A 5 ml yeast culture was grown in SCD (-Leu) at 30 °C until an OD_600_ of 0.8. Subsequently, the culture was diluted in the same medium to an OD_600_ of 0.1 and was grown at 37 °C until an OD_600_ of 0.8 was reached. Total cell extracts were prepared by alkaline lysis. In this method, 1 ml of the cell culture was centrifuged (5 min, 800 x g) and the cell pellet was resuspended in 100 μl ice-cold lysis buffer (0.2 M NaOH, 0.5% (v/v) β-mercaptoethanol). After 30 min incubation on ice, 2 μl of 6 N HCl and 50 μl of 3X Laemmli buffer were added and the samples were incubated at 95 °C for 10 min. The accumulation of the precursor form of Hsp60 was analyzed by SDS-PAGE of the cell extracts (5 μl) using a 12% acrylamide gel followed by immunoblot with antibodies against Hsp60. Blots were visualized by the Odyssey Infrared Imaging System (LI-COR).

### *In vitro* cross-linking of Tim23_IMS_ with Tim50_IMS_

Tim50_IMS_ (10 µM) was incubated with or without Tim23_IMS_ (5 µM) in buffer containing 20 mM HEPES/NaOH, pH 7.4, 5% glycerol, 200 mM NaCl, 5 mM MgCl_2_ and 50 mM KCl, for 10 min at room temperature. Cross-linking reactions were performed by addition of 1 mM DSS for 1 h at room temperature. Cross-linking adducts were analyzed by SDS-PAGE followed by CBB staining or by immunoblotting with antibodies against Tim23 or Tim50.

### Miscellaneous

Pull down experiments and protein purification were carried out as previously described^10,20^. Standard protocols were used for SDS PAGE and Western blot analysis using the Odyssey Infrared Imaging System (Licor).

## Supplementary information

Supplementary

## Data Availability

The datasets generated during and/or analyzed during the current study are available from the corresponding author on reasonable request.

## References

[1] Mokranjac D, Neupert W (2010). The many faces of the mitochondrial TIM23 complex. Biochim Biophys Acta..

[2] Schulz C, Schendzielorz A, Rehling P (2015). Unlocking the presequence import pathway. Trends Cell Biol.

[3] Song J, Tamura Y, Yoshihisa T, Endo T (2014). A novel import route for an N-anchor mitochondrial outer membrane protein aided by the TIM23 complex. EMBO Rep.

[4] Wenz LS (2014). The presequence pathway is involved in protein sorting to the mitochondrial outer membrane. EMBO Rep.

[5] Sinzel M (2016). Mcp3 is a novel mitochondrial outer membrane protein that follows a unique IMP-dependent biogenesis pathway. EMBO Rep.

[6] Bauer MF, Sirrenberg C, Neupert W, Brunner M (1996). Role of Tim23 as voltage sensor and presequence receptor in protein import into mitochondria. Cell.

[7] Truscott KN (2001). A presequence- and voltage-sensitive channel of the mitochondrial preprotein translocase formed by Tim23. Nat Struct Biol.

[8] Martinez-Caballero S, Grigoriev SM, Herrmann JM, Campo ML, Kinnally KW (2007). Tim17p regulates the twin pore structure and voltage gating of the mitochondrial protein import complex TIM23. J Biol Chem.

[9] Pareek G, Krishnamoorthy V, D’Silva P (2013). Molecular insights revealing interaction of Tim23 and channel subunits of presequence translocase. Mol Cell Biol.

[10] Demishtein-Zohary K, Marom M, Neupert W, Mokranjac D, Azem A (2015). GxxxG motifs hold the TIM23 complex together. The FEBS journal.

[11] van der Laan M (2006). A role for Tim21 in membrane-potential-dependent preprotein sorting in mitochondria. Curr Biol.

[12] Wiedemann, N., van der Laan, M., Hutu, D. P., Rehling, P. & Pfanner, N. Sorting switch of mitochondrial presequence translocase involves coupling of motor module to respiratory chain. *J Cell Biol* **179** (2007). 10.1083/jcb.200709087 PMC2140023 18070913

[13] Gebert M (2012). Mgr2 promotes coupling of the mitochondrial presequence translocase to partner complexes. J Cell Biol.

[14] Marom M, Azem A, Mokranjac D (2011). Understanding the molecular mechanism of protein translocation across the mitochondrial inner membrane: still a long way to go. Biochim Biophys Acta.

[15] Schulz C (2011). Tim50’s presequence receptor domain is essential for signal driven transport across the TIM23 complex. J Cell Biol.

[16] Qian X (2011). Structural basis for the function of Tim50 in the mitochondrial presequence translocase. J Mol Biol.

[17] Lytovchenko O (2013). Signal recognition initiates reorganization of the presequence translocase during protein import. EMBO J.

[18] Waegemann K, Popov-Celeketic D, Neupert W, Azem A, Mokranjac D (2015). Cooperation of TOM and TIM23 complexes during translocation of proteins into mitochondria. J Mol Biol.

[19] Malhotra K (2017). Cardiolipin mediates membrane and channel interactions of the mitochondrial TIM23 protein import complex receptor Tim50. Sci Adv.

[20] Gevorkyan-Airapetov L (2009). Interaction of Tim23 with Tim50 Is essential for protein translocation by the mitochondrial TIM23 complex. J Biol Chem.

[21] Meinecke M (2006). Tim50 maintains the permeability barrier of the mitochondrial inner membrane. Science.

[22] Li J, Sha B (2015). The structure of Tim50(164-361) suggests the mechanism by which Tim50 receives mitochondrial presequences. *Acta crystallographica*. Section F, Structural biology communications.

[23] Rahman B, Kawano S, Yunoki-Esaki K, Anzai T, Endo T (2014). NMR analyses on the interactions of the yeast Tim50 C-terminal region with the presequence and Tim50 core domain. FEBS Lett.

[24] Tamura Y (2009). Tim23-Tim50 pair coordinates functions of translocators and motor proteins in mitochondrial protein import. J Cell Biol.

[25] Bajaj R, Jaremko L, Jaremko M, Becker S, Zweckstetter M (2014). Molecular Basis of the Dynamic Structure of the TIM23 Complex in the Mitochondrial Intermembrane Space. Structure.

[26] Geissler A (2002). The mitochondrial presequence translocase: an essential role of Tim50 in directing preproteins to the import channel. Cell.

[27] Alder NN, Sutherland J, Buhring AI, Jensen RE, Johnson AE (2008). Quaternary structure of the mitochondrial TIM23 complex reveals dynamic association between Tim23p and other subunits. Mol Biol Cell.

[28] Yamamoto H (2002). Tim50 is a subunit of the TIM23 complex that links protein translocation across the outer and inner mitochondrial membranes. Cell.

[29] de la Cruz L, Bajaj R, Becker S, Zweckstetter M (2010). The intermembrane space domain of Tim23 is intrinsically disordered with a distinct binding region for presequences. Protein Sci.

[30] Mokranjac D (2009). Role of Tim50 in the transfer of precursor proteins from the outer to the inner membrane of mitochondria. Mol Biol Cell.

[31] Glaser F (2003). ConSurf: identification of functional regions in proteins by surface-mapping of phylogenetic information. Bioinformatics.

