# Supplementary material for: A mutagenesis analysis of Tim50, the major receptor of the TIM23 complex, identifies regions that affect its interaction with Tim23
Source: Sci Rep. 2019 Feb 14;9:2012. doi: 10.1038/s41598-018-38353-1 (PMC6375917; doi:10.1038/s41598-018-38353-1)
Supplement: Supplementary file 1 — Supplementary [file 41598_2018_38353_MOESM1_ESM.docx]

A mutagenesis analysis of Tim50, the major receptor of the TIM23 complex, identifies regions that affect its interaction with Tim23

Dana Dayan^1^, May Bandel^1^, Umut Günsel^2^, Inbal Nussbaum^1^, Gali Prag^1^, Dejana Mokranjac^2^, Walter Neupert^3^ and Abdussalam Azem^*, 1^

^1^Department of Biochemistry and Molecular Biology, The George S. Wise Faculty of Life Sciences, Tel Aviv University, Tel Aviv 69978, Israel

^2^Biomedical Center Munich - Physiological Chemistry, LMU Munich, 82152 Martinsried, Germany

^3^Max Planck Institute of Biochemistry, 82152 Martinsried, Germany

^*^Correspondence to Abdussalam Azem: [azema@tauex.tau.ac.il](mailto:azema@tauex.tau.ac.il) Fax: +972-3-6406834 Tel: +972-3-6409007

**Supplementary Data**

**Table S1. List of primers used in site-directed mutagenesis**

| **Primer name** | **Primer Sequence 5’… 3’** |
| --- | --- |
| V274E | GTTTACAAAGACGGTGAGCACATTAAGGATCTG |
|  | CAGATCCTTAATGTGCTCACCGTCTTTGTAAAC |
| R218G | GTTGGAGAACGGCCAAAGGACCTGGTGCTGACTAC |
|  | GTAGTCAGCACCAGGTCCTTTGGCCGTTCTCCAAC |
| L202M | TGGAAGATTTTATGGTTCATTCT |
|  | AGAATGAACCATAAAATCTTCCA |
| P175H | CTACTTCCAAGAGCCACATTTCCCTGATTTACTAC |
|  | GTAGTAAATCAGGGAAATGTGGCTCTTGGAAGTAG |
| D278V | GACGGTGTGCACATTAAGGTACTGTCAAAATTGAATAGAG |
|  | CTCTATTCAATTTTGACAGTACCTTAATGTGCACACCGTC |
| R339S | CAACAAACCAAGGATGTTAGCCCAATCTTGAACAGCTTTG |
|  | CAAAGCTGTTCAAGATTGGGCTAACATCCTTGGTTTGTTG |
| N283Y | GGATCTGTCAAAATTGTACAGAGATTTGAG |
|  | CTCAAATCTCTGTACAATTTTGACAGATCC |
| D293N | GTAAAGTAATCATTATTAACACTGACCCTAACAG |
|  | CTGTTAGGGTCAGTGTTAATAATGATTACTTTAC |
| E439G | CTGAAAGACTATGTTGGCGGTAACTTGCCTTCGC |
|  | GCGAAGGCAAGTTACCGCCAACATAGTCTTTCAG |
| A221D | CAAAAGACCTGGTGATGACTACTTCTTGGG |
|  | CCCAAGAAGTAGTCATCACCAGGTCTTTTG |
| M242K | ATCCAACTATAAGATGTACTCTGAC |
|  | GTCAGAGTACATCTTATAGTTGGAT |
| D337V | CACTCAACAAACCAAGGTTGTTAGACCAATCTTG |
|  | CAAGATTGGTCTAACAACCTTGGTTTGTTGAGTG |
| L194H | CCAAAGGCCATTAACTCATGTTATCACATTGGAAG |
|  | CTTCCAATGTGATAACATGAGTTAATGGCCTTTGG |
| E415D | CATGAAGATGATTGAGGATGAAAAGGAAAAAATTAG |
|  | CTAATTTTTTCCTTTTCATCCTCAATCATCTTCATG |

**Figure S1**


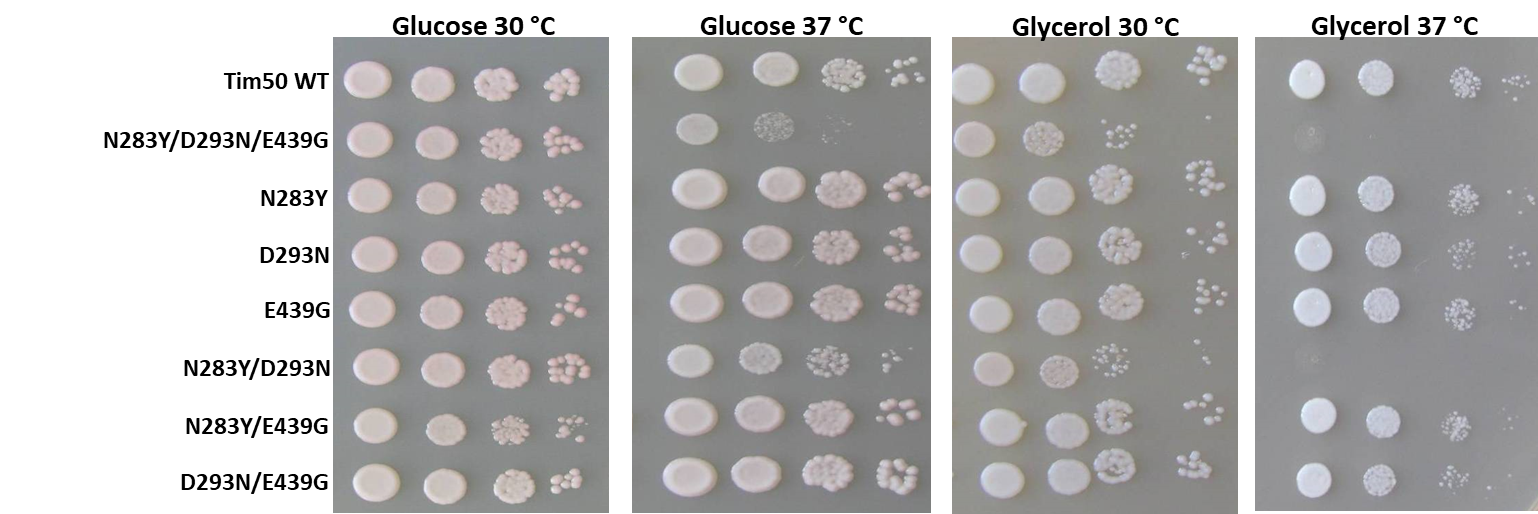


**Figure S1**. **Growth analysis of mutant #4 and the mutants derived from it.** Serial dilutions of Tim50 WT cells and mutants originated from mutant #4 were grown for 5 days at the indicated temperature on SCD (-Leu) and SCG plates.

**Figure S2**

**
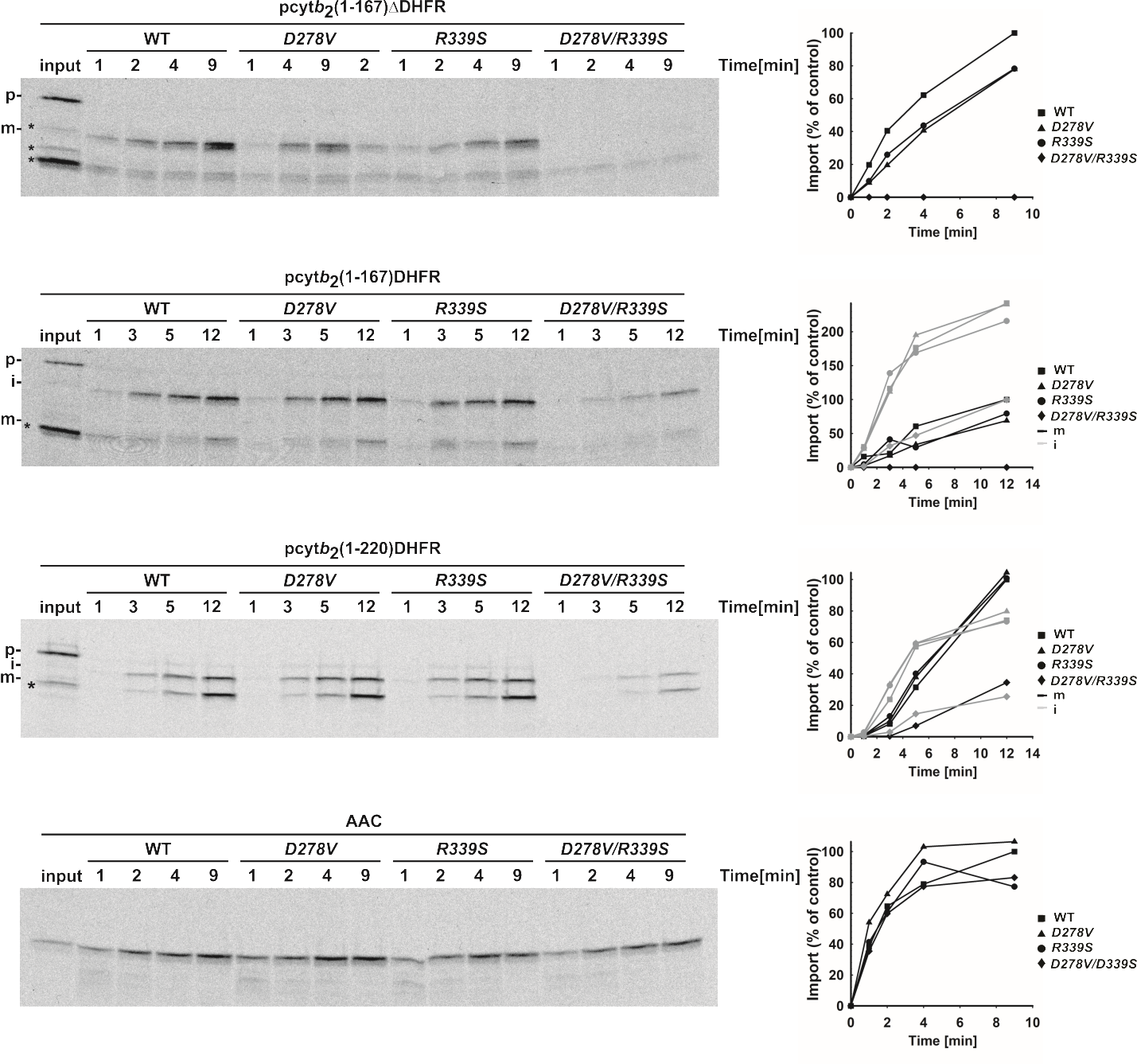
**

**
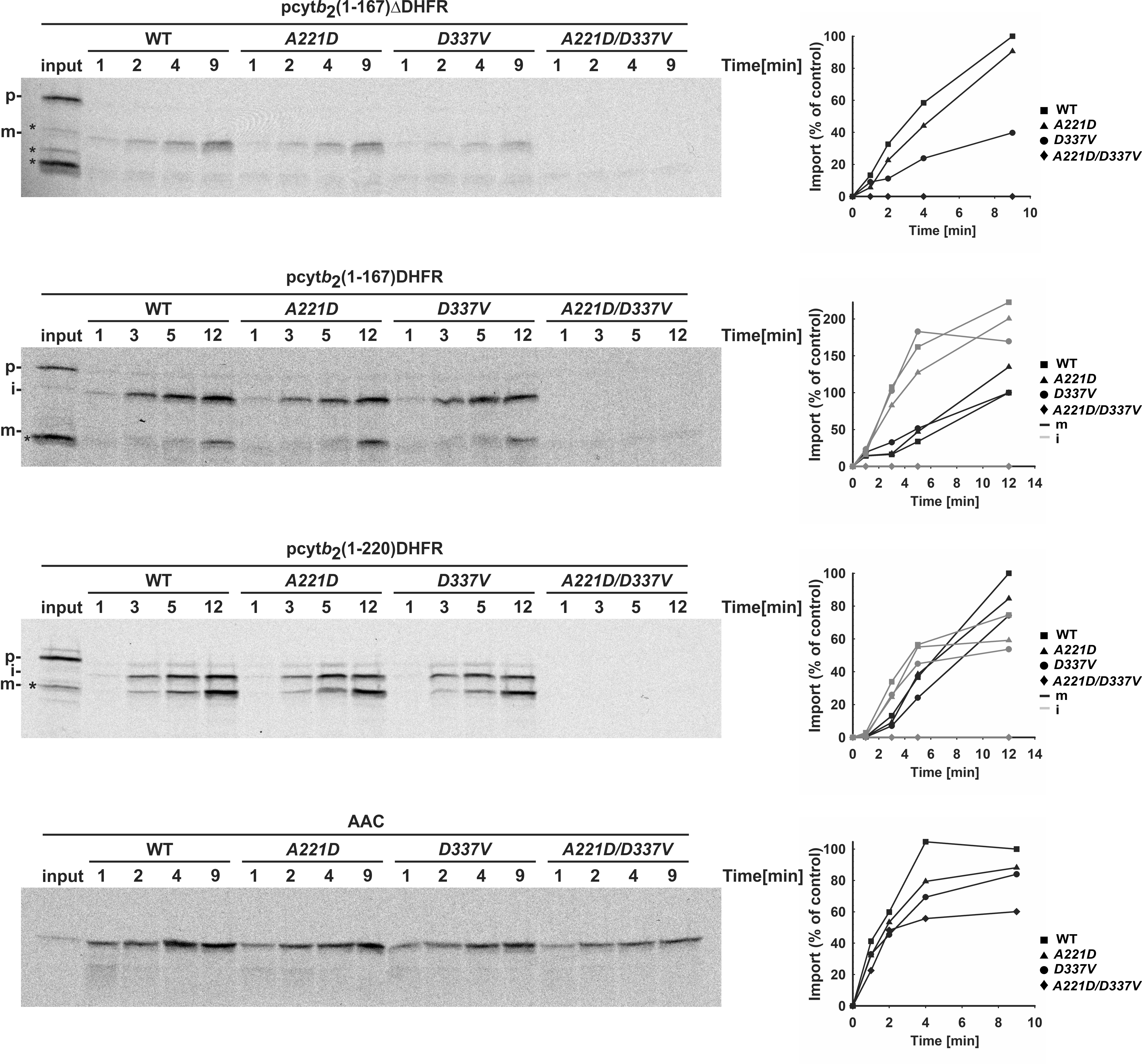

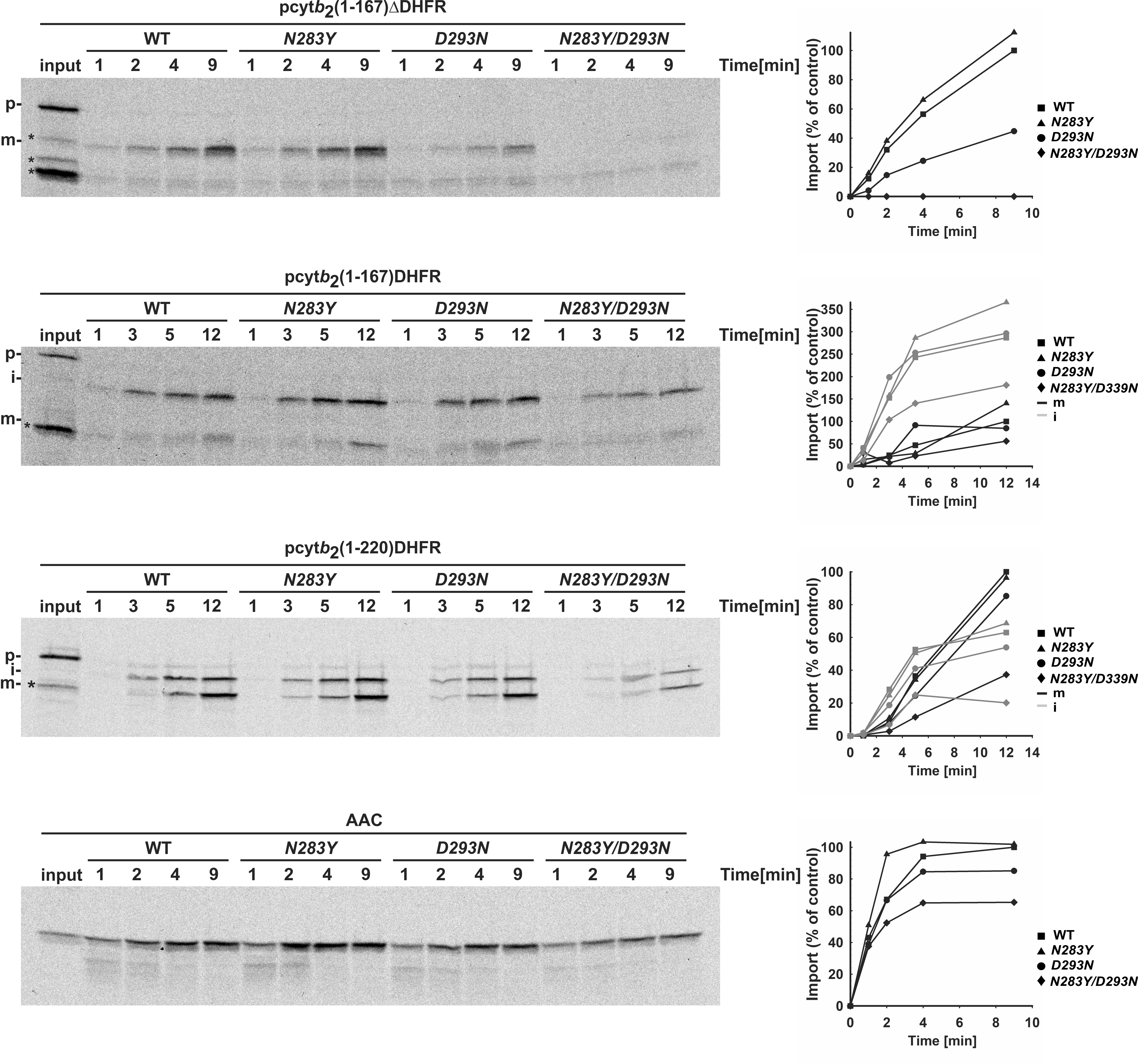
**

**Figure S2**. **Import of precursors into isolated mitochondria.** Yeast strains containing the indicated N-terminally His-tagged versions of Tim50 were grown in lactate medium containing 0.1% glucose at 24°C. Isolated mitochondria were preincubated for 30 min at 37°C before shifting the temperature to 25°C for *in vitro* import of various ^35^S-Met labelled mitochondrial precursors. Three TIM23-dependent precursors were used, a matrix targeted pcyt*b*_2_(1-167)ΔDHFR and two laterally sorted precursor proteins pcyt*b*_2_(1-167)DHFR and pcyt*b*_2_(1-220)DHFR. These three commonly used precursor proteins consist of the depicted segments of yeast cytochrome *b_2_* fused to full length mouse dihydrofolate reductase (DHFR). The first 220 residues of cyt *b_2_* contain the presequence, the lateral sorting signal and the entire heme-binding domain (HBD) of cyt *b_2_*. During import, the presequence of pcyt*b*_2_(1-220)DHFR is removed in the mitochondrial matrix to generate intermediate (i) form. The lateral sorting signal then stops the translocation into the matrix, the protein is inserted into the inner membrane and the second processing step occurs on the intermembrane space side of the inner membrane to generate soluble, mature (m) form of the protein. The difference between pcyt*b*_2_(1-220)DHFR and pcyt*b*_2_(1-167)DHFR is that the former has the entire HBD of cyt *b_2_* rendering the import process dependent on the ATPase activity of the import motor of the TIM23 complex. In contrast, pcyt*b*_2_(1-167)DHFR is imported in an ATP-independent manner due to removal of part of HBD. In pcyt*b*_2_(1-167)ΔDHFR the lateral sorting signal has been deleted, leading to ATP-dependent translocation of the entire protein into the matrix. ^35^S-radiolabeled AAC (ATP/ADP carrier) was used as a control for TIM23-independent import. At the indicated time points, samples were diluted into ice-cold buffer containing valinomycin to quench further import. Samples were treated with Proteinase K to degrade non-imported proteins and subsequently analyzed by SDS-PAGE followed by transfer onto nitrocellulose membrane and autoradiography (left panels). Image J software was used to quantify import reactions (right panels). The intensity of the mature band in the longest time point upon import into WT mitochondria was set to 100%. i, intermediate and m, mature forms of imported precursors.

**Figure S3**

**
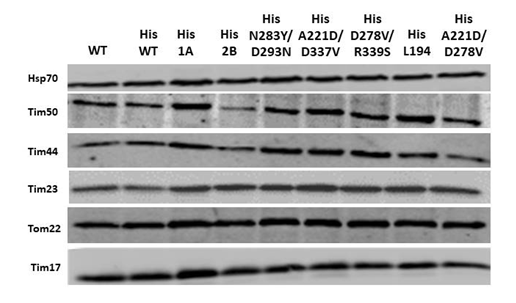
**

**Figure S3. Endogenous levels of mitochondrial proteins.** Mitochondria (5µg protein) isolated from WT and mutant cells were analyzed by SDS-PAGE followed by immunoblotting with the indicated antibodies.
